# Hop-on hop-off: importin-α-guided tours to the nucleus in innate immune signaling

**DOI:** 10.3389/fpls.2013.00149

**Published:** 2013-05-21

**Authors:** Lennart Wirthmueller, Charlotte Roth, Mark J. Banfield, Marcel Wiermer

**Affiliations:** ^1^Department of Biological Chemistry, John Innes Centre, Norwich Research ParkNorwich, UK; ^2^Albrecht-von-Haller-Institute for Plant Sciences, Department of Plant Cell Biology, Georg-August-University GöttingenGöttingen, Germany

**Keywords:** importin-α, nuclear protein import, nucleocytoplasmic transport, *Arabidopsis*, innate immunity

## Abstract

Nuclear translocation of immune regulatory proteins and signal transducers is an essential process in animal and plant defense signaling against pathogenic microbes. Import of proteins containing a nuclear localization signal (NLS) into the nucleus is mediated by nuclear transport receptors termed importins, typically dimers of a cargo-binding α-subunit and a β-subunit that mediates translocation through the nuclear pore complex. Here, we review recent reports of importin-α cargo specificity and mutant phenotypes in plant- and animal–microbe interactions. Using homology modeling of the NLS-binding cleft of nine predicted *Arabidopsis *α-importins and analyses of their gene expression patterns, we discuss functional redundancy and specialization within this transport receptor family. In addition, we consider how pathogen effector proteins that promote infection by manipulating host cell nuclear processes might compete with endogenous cargo proteins for nuclear uptake.

## HOP-ON HOP-OFF: IMPORTIN-MEDIATED NUCLEAR PROTEIN IMPORT

In eukaryotic cells, nuclear transport receptors (NTRs) of the importin-α family recognize and bind to canonical nuclear localization signal (NLS)-containing cargo proteins in the cytoplasm and link them to importin-β, the NTR that facilitates passage of the ternary complex through the nuclear pore complex (NPC) into the nucleus. Cargos may contain one (monopartite) or two (bipartite) NLS sequence motifs and directional binding to and release from the importin-α/β heterodimer is imposed by the nucleotide-binding state of Ran, a small guanosine-5^′^-triphosphatase (GTPase) that cycles between GTP-bound nuclear and guanosine-5^′^-diphosphate (GDP)-bound cytoplasmic states (Terry et al., 2007; Meier and Somers, 2011). The RanGDP-RanGTP gradient across the nuclear envelope (NE) is generated by the asymmetric distribution of two regulators, RanGAP in the cytoplasm and RanGEF in the nucleus that is associated with chromatin and drives nuclear cargo release upon binding of RanGTP to importin-β. After dissociation of the import complex and cargo delivery into the nucleus, importin-β bound to RanGTP is recycled to the cytoplasm, whereas importin-α interacts with the RanGTP-bound export receptor CAS for recycling of cargo-free importin-α back to the cytoplasm. In the cytoplasm, RanGAP stimulates GTP hydrolysis on Ran to release the importins for another round of import (Stewart, 2007).

**FIGURE 1:**
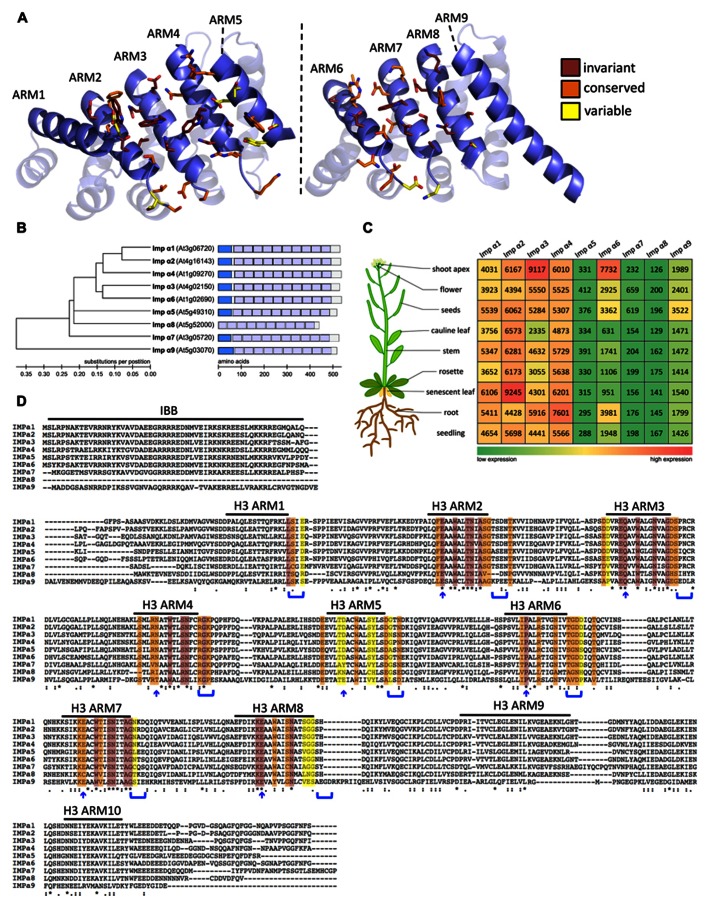
**Expression profile and sequence comparison of *Arabidopsis* importins α1-9**. **(A)** Homology model of the ARM repeat domain of *Arabidopsis *importins α1-9 based on the structure of rice importin-α1a (RCSB identifier 4B8J, Chang et al., 2012). Left image: major NLS binding site. Right image: minor NLS binding site. Amino acids that are likely to contribute to the NLS binding sites are shown in stick representation. The color code indicates the level of conservation in *Arabidopsis *α-importins. **(B)** Phylogenetic tree constructed using neighbor joining in Molecular Evolutionary Genetics Analysis (MEGA) v4.0 (Tamura et al., 2007). Importin-α9 was used to root the tree. Scale bar represents amino acid substitutions per position. Schematic representation: The different protein domains are depicted as boxes within the full length protein sequence. Importin-β-binding domains are shown in dark blue and the ten Armadillo repeat domains are shown in light blue. Scale bar shows number of amino acids. **(C)** Gene expression data were gathered from the Genevestigator database (https://www.genevestigator.com/gv/; Hruz et al., 2008). Data referring to whole tissues were chosen for comparison of expression levels. Numbers represent linear signal intensity values of the given gene in the indicated tissues. Heat map indicates low signal intensity (green) to high signal intensity (red). **(D)** Multiple sequence alignment of full-length protein sequences performed using ClustalW2 (http://www.ebi.ac.uk/Tools/msa/clustalw2/; Larkin et al., 2007). Color code for conservation as in A. Blue arrows and parenthesis indicate candidate amino acids that are predicted to contribute to the NLS binding sites based on analysis of yeast, mouse, and human α-importins (Marfori et al., 2012). Variations in these motifs are likely to determine specificity of α-importins for NLS binding.

α-importins typically consist of an N-terminal auto-inhibitory importin-β-binding (IBB) domain followed by a series of ten armadillo (ARM) repeats that form the NLS-binding cleft (Goldfarb et al., 2004; **Figures 1A,B**). The flexible IBB domain not only connects importin-α to importin-β but also contains a cluster of basic amino acids that competes with NLS-cargos for binding to the ARM-repeat domain of importin-α. Thus, the IBB domain is involved in regulating both formation of the trimeric import complex in the cytoplasm and release of cargo in the nucleus after the IBB domain is freed from importin-β by RanGTP (Görlich et al., 1996a; Kobe, 1999; Stewart, 2007). Following cargo release in the nucleus α-importin is exported to the cytoplasm by a complex of the export carrier CAS and RanGTP (Goldfarb et al., 2004; Matsuura and Stewart, 2004).

Stimulus-induced nuclear translocation and/or accumulation of signaling molecules and transcriptional regulators are essential for the coordinated relay of defense signals in both plant and animal innate immune responses to microbial pathogens. Inside the nucleus, these signals direct the expression of defense-related genes. In addition, it has become increasingly evident that not only do host resistance components show dynamic partitioning between the cytoplasm and the nucleus, but also that a significant number of animal and plant pathogen virulence factors exploit host cell nuclear import pathways to act directly within the nucleus and promote disease. In this review, we provide an overview of recent studies reporting importin-α cargo selectivity in animal and plant innate immunity and discuss potential promiscuity within the *Arabidopsis* import receptor family. We also consider how microbial virulence factors may hijack the nuclear import machinery to manipulate host cell nuclear processes.

## IMPORTIN-α PARALOGS IN *Arabidopsis thaliana*

Although the *Saccharomyces cerevisiae* genome encodes only a single importin-α (Yano et al., 1992), several paralogs have been reported in most higher eukaryotes – seven in humans, six in mouse, three in *Drosophila*, five in rice, and nine in *Arabidopsis *(Ouyang et al., 2007; Ratan et al., 2008; Hu et al., 2010; Kelley et al., 2010; Merkle, 2011). Conceivably, expansion of the *importin-α* gene family in multicellular eukaryotes reflects adaptation toward a more complex regulation of nuclear import. Several mammalian *importin-α *paralogs show tissue-specific expression patterns (Köhler et al., 1997; Tsuji et al., 1997; Yasuhara et al., 2007), and nuclear import of some cargo proteins is preferentially mediated by specific importin-α adapters (Miyamoto et al., 1997; Nadler et al., 1997; Köhler et al., 1999; Melén et al., 2003; Quensel et al., 2004). In *Arabidopsis importin-α1-4*, *α6*, and *α9* are ubiquitously expressed (**Figure 1C**). However, there is controversy from different profiling techniques regarding the levels and tissue-specificity of *importin-α5*, *α7*, and *α8 *expression (Meyers et al., 2004; Bhattacharjee et al., 2008; Hruz et al., 2008; Huang et al., 2010). For example, although Huang et al. (2010) report specific expression of *importin-α8* in rosette/cauline leaves and flowers, a search for genes regulated by the male germ line-specific transcription factor (TF) DUO1 suggests that *importin-α8* is a DUO1 target gene that is specifically expressed in the male germ line (Borg et al., 2011). These data indicate that importin-α8 may have a distinct function during pollen development. Notably, importin-α8 does not have an IBB domain (**Figure 1B**) suggesting that it lacks both the capacity to bind importin-β and the auto-inhibitory mechanisms that are conserved in the other α-importins. Therefore, it remains to be tested if importin-α8 can function as a NTR and whether the loss of the IBB domain is a consequence of specialization in pollen development.

The comparably high number of α-importins in *Arabidopsis* can only partially be rationalized by tissue-specific expression of single paralogs. Alternatively, multiple paralogs might have evolved to transport specific cargos. Indeed, the NLS from the rice COP1 protein binds *in vitro *the two rice importins α1a and α1b, but not importin-α2 (Jiang et al., 2001). This, and other examples outlined below, provides evidence for cargo specificity of α-importins and it appears likely that higher eukaryotes are equipped with an array of α-importins that accumulate to different levels and exhibit different affinities for distinct cargos. Transcriptional and post-translational regulation of importin-α protein levels in response to environmental stimuli would constitute a flexible system to alter nuclear import kinetics and specificities in changing environments.

## SEQUENCE DIVERSITY IN *Arabidopsis* α-IMPORTINS

Resolved crystal structures of α-importins from yeast, human, mouse, and rice revealed strong structural conservation of the ARM repeat domains that form the NLS binding sites (Conti et al., 1998; Kobe, 1999; Fontes et al., 2003; Chang et al., 2012). ARM repeats from yeast, human, and mouse α-importins can be superimposed with a root mean square deviation of less than 1.8Å and amino acids that contribute to the NLS binding sites occupy very similar positions in these structures. We used homology modeling to characterize conservation of the NLS binding site among the nine *Arabidopsis* α-importins. As in α-importins from other species, a conserved array of Trp/Asn pairs protruding from the third helix of the ARM repeats (H3) forms the core of the major and minor NLS binding sites in *Arabidopsis *α-importins (**Figure 1A**). Previous comparative analysis revealed that major determinants of specificity are (i) the amino acid positioned three residues upstream of the conserved Trp, and (ii) residues that constitute the loops connecting the H3 and H1 helices (Marfori et al., 2012). Notably, the Trp/Asn array at the minor NLS binding site is not entirely conserved in plant α-importins (**Figure 1D** and **Table 1**). As some plant NLSs specifically bind to the minor NLS binding site (Chang et al., 2012) it will be interesting to test whether these divergent amino acids determine binding to specific NLSs.

**Table 1:** Some plant α-importins diverge from the otherwise conserved pattern of amino acids protruding from ARM H3 helices that form the core of the NLS binding sites. The amino acid pairs denoted as consensus sequence (column two) are conserved in α-importins from yeast, human, mouse, and *Drosophila*, as well as the remaining α-importins from *Arabidopsis* and rice. Amino acids in blue bold font indicate divergence from the consensus sequence whereas “cons.” indicates conservation of the consensus sequence.

ARM repeat	Consensus sequence	At importinα5	At importinα8	At importinα9	Os importin Os07g48880	Os importinα2
ARM2	Trp/Asn	cons.	cons.	cons.	cons.	cons.
ARM3	Trp/Asn	cons.	cons.	cons.	cons.	cons.
ARM4	Trp/Asn	cons.	cons.	cons.	cons.	cons.
ARM5	Trp/Tyr	Trp/Asn	Met/His	cons.	cons.	cons.
ARM6	Arg/Asn	cons.	Leu/Ala	cons.	Thr/Arg	cons.
ARM7	Trp/Asn	cons.	cons.	cons.	Leu/Asn	cons.
ARM8	Trp/Asn	cons.	cons	Tyr/Asn	cons.	Tyr/Asn

## IMPORTIN-α CARGO SPECIFICITY IN ANIMAL IMMUNE RESPONSES

Both animal and plant innate immune systems have evolved pattern recognition receptors (PRRs) to detect microbe-associated molecular patterns (MAMPs) and defend against pathogens (Nürnberger et al., 2004; Ausubel, 2005). In addition to MAMP detection, the plant innate immune system also imparts pathogen-specific recognition via nucleotide-binding/leucine-rich repeat immune sensors (NLRs) that detect the actions of isolate-specific pathogen virulence factors, termed effectors (Jones and Dangl, 2006). In contrast, animal NLRs detect MAMPs inside host cells (Kanneganti et al., 2007a; Ronald and Beutler, 2010; Maekawa et al., 2011). Activation of both NLRs and PRRs initiates signaling cascades that convey the biotic stress stimulus into the host cell nucleus to alter defense gene expression. Thus, stimulus-induced changes in the NPC permeability of signal transducers, immune and transcriptional regulators represent an important mechanism for controlling defense-associated gene expression.

Changes in nuclear translocation rates are often achieved via post-translational protein modifications leading to conformational changes that expose or conceal NLSs or nuclear export sequences (NESs). For example, gene expression changes in mammalian innate immunity are largely governed by the induced nuclear translocation of the NF-κB family of Rel-type TFs. Nuclear accumulation of NF-κB is controlled by its association with IκB proteins. Depending on the type of IκB, these proteins either sequester NF-κB in the cytoplasm by masking its NLS, or prevent its ability to bind to chromatin due to a strong NES in IκB that directs dominant nuclear export over nuclear import (Johnson et al., 1999; Huang et al., 2000; Malek et al., 2001). Signal-dependent phosphorylation by IκB-kinase targets IκB for proteolysis, thereby allowing NF-κB nuclear import to activate defense gene expression. In human cells, the closely related importins α3 and α4 are the two main isoforms responsible for nuclear import of NF-κB p50/p65 heterodimers following IκB degradation. Whereas the major NLS binding site of importin-α3 binds to p50, the minor NLS binding site mediates association with p65 (Fagerlund et al., 2005).

Innate immune responses in *Drosophila *are also controlled at the level of nuclear transport. Upon activation of the Toll signaling cascade, NF-κB/Rel-type TFs translocate to the nucleus in a process that is dependent on nuclear transport factor-2 (NTF-2), an essential component of nuclear trafficking that acts as nuclear import receptor for RanGDP to replenish the nuclear Ran pool (Ribbeck et al., 1998; Smith et al., 1998; Bhattacharya and Steward, 2002). Whether NTF-2 directly binds Rel proteins or indirectly affects their nuclear import rates by regulating the function of *Drosophila* α-importins remains to be determined.

Like NF-κB, signal transducers and activators of transcription (STAT) proteins are a family of latent cytoplasmic TFs, consisting of seven members in mammals. Upon cytokine activation of the canonical STAT-signaling pathway, tyrosine phosphorylation induces STAT homo- or hetero-dimerization and subsequent importin-α-dependent nuclear import (Lim and Cao, 2006). Activated STAT1 homodimers and STAT1/STAT2 heterodimers interact with importin-α5 (Melén et al., 2001; Fagerlund et al., 2002) whereas RNAi-mediated silencing of *importin-α3* but not of other tested *importin-α* family members impairs nuclear translocation of STAT3, but not of STAT1 (Liu et al., 2005). This indicates that different α-importins can have distinct STAT protein binding preferences.

Further examples of vertebrate immune regulatory proteins that contain NLSs and can shuttle into the nucleus are the NLRs CIITA and NLRC5. Both these proteins function through association with DNA-binding proteins to regulate MHC class II and class I gene expression, respectively (Spilianakis et al., 2000; Cressman et al., 2001; Meissner et al., 2012). Correlating potential importin-α binding specificities for CIITA and NLRC5 remains to be determined.

## IMPORTIN-α CARGO SPECIFICITY IN PLANT INNATE IMMUNITY

In rice, the intracellular kinase domain of the PRR XA21 carries a functional NLS and translocates to the nucleus after cleavage from the activated receptor, probably to modulate transcription (Park and Ronald, 2012). Also, several NLRs exhibit nucleocytoplasmic partitioning, including *Arabidopsis* RPS4, snc1 and RRS1-R, tobacco N, barley MLAs, and potato Rx (Deslandes et al., 2003; Burch-Smith et al., 2007; Shen et al., 2007; Wirthmueller et al., 2007; Cheng et al., 2009; Slootweg et al., 2010; Tameling et al., 2010). Except for MLA and Rx, these proteins possess predicted NLSs and it appears that mono- or bipartite NLSs are widespread among *Arabidopsis* NLRs (Shen and Schulze-Lefert, 2007; Caplan et al., 2008; Liu and Coaker, 2008). However, experimental proof for the function of these motifs has only been provided for RPS4 (Wirthmueller et al., 2007) and it is not understood how nucleocytoplasmic partitioning of these immune sensors is regulated.

Besides NLRs, the dynamic translocation of several plant immune regulatory proteins is a key factor in defense pathway regulation. In healthy *Arabidopsis* cells, the transcriptional co-activator NPR1 is retained partially in the cytoplasm as a homo-oligomeric complex. Changes in the cell’s redox potential, induced by the defense hormone salicylic acid, promotes release of NPR1 monomers and their nuclear accumulation, presumably via exposure of an obscured NLS (Kinkema et al., 2000; Mou et al., 2003; Tada et al., 2008). A negative regulator of cell death, the *Arabidopsis *zinc finger protein LSD1, antagonizes transcriptional activity of the nucleocytoplasmic shuttling leucine-zipper TF, bZIP10, by sequestering bZIP10 in the cytoplasm. Dissociation in response to pathogens is thought to unmask the NLS of bZIP10, permitting its nuclear translocation and expression of target genes (Kaminaka et al., 2006). Another report suggests that LSD1 itself localizes to nuclei, as *Pisum sativum *LSD1 is nuclear when transiently expressed in *Arabidopsis* protoplasts. *Ps*LSD1 nuclear localization is mediated by its zinc finger motifs that interact with several *Arabidopsis *α-importins and may constitute a novel NLS (He et al., 2011). The cell death pathway repressed by LSD1 depends on the activities of EDS1 and PAD4, two key regulators of basal resistance and immunity triggered by Toll interleukin-1 receptor (TIR)-type NLRs (Aarts et al., 1998; Feys et al., 2001; Wiermer et al., 2005). EDS1 harbors a predicted NLS and NES and forms dynamic nucleocytoplasmic complexes with PAD4 and SAG101, yet NTR binding-specificities responsible for nuclear targeting remain elusive (Feys et al., 2005; Garcia et al., 2010).

Evidence of importin-α cargo specificity in plants comes from a report by Kanneganti et al. (2007b). Silencing of *Nicotiana benthamiana importin-α1* and *α2* inhibits nuclear targeting of the transiently expressed *Phytophthora infestans *effectors Nuk6 and Nuk7 while nuclear import of Nuk12 is unaffected.

Constitutive immune signaling induced by a point mutation in SNC1, an *Arabidopsis *TIR-type NLR, is partially suppressed by mutations in *importin-α3* (Palma et al., 2005). A pool of active snc1 protein is found in nuclei and auto-immunity is abolished by a snc1-NES fusion (Cheng et al., 2009). Overexpression of GFP-tagged SNC1-4 (a mutant version of snc1-1) in wild type *Arabidopsis *protoplasts results in an entirely nuclear accumulation of the fusion protein, while the same construct is nucleocytoplasmic in protoplasts lacking importin-α3 (Zhu et al., 2010). Although this makes importin-α3 a candidate NTR of SNC1-4 it remains to be tested whether SNC1-4 binds importin-α3 directly. Alternatively, importin-α3 may be required for nuclear import of signaling components activated by snc1. Partial suppression of the *snc1-1* phenotype by knock-out of *importin-α3* indicates that other α-importins might work redundantly with importin-α3 in *snc1*-triggered immunity.

A knock-out of *Arabidopsis importin-α4* results in a *rat *(resistant to *Agrobacterium* transformation) phenotype (Bhattacharjee et al., 2008). Transformation by *Agrobacterium* requires active nuclear import of the transfer DNA/protein complex (T-complex). Two *Agrobacterium* effectors, VirD2 and VirE2 are essential for plant transformation and both proteins carry NLSs, providing a molecular link between the T-complex and the host’s nuclear import machinery (Gelvin, 2010; Pitzschke and Hirt, 2010). Although VirE2 and VirD2 can interact with several *Arabidopsis *α-importins, only a knock-out of *importin-α4* impairs host transformation (Bhattacharjee et al., 2008). Significantly, the *rat *phenotype is not only complemented by *importin-α4 *overexpression but also by overexpression of six other *Arabidopsis α-importins*. This suggests that although importin-α4 is the most relevant NTR for the T-complex other α-importins can complement loss of importin-α4 function when their protein levels are increased. These results are in agreement with findings in yeast which show that nuclear import of different NLSs, with varying affinities for importin-α, is largely governed by the rate of NLS/importin-α complex formation (Riddick and Macara, 2005; Hodel et al., 2006; Timney et al., 2006). Thus, nuclear import rates can be elevated by either increasing protein levels of the cargo or importin-α, or by increasing the affinity of the NLS for the NTR.

## HOLD ON TIGHT - NUCLEAR PATHOGEN EFFECTORS AND THE IMPORTIN-α/NLS AFFINITY CONTROVERSY

Notably, the “optimal” binding affinity of a NLS for importin-α is still controversial. Several *in vitro* studies reported dissociation constants in the low nanomolar range based on indirect affinity measurements (Hodel et al., 2001; Timney et al., 2006; Kosugi et al., 2008). Two other studies determined NLS/importin-α affinities *in vitro* by isothermal titration calorimetry and found K_d_ values of ~3 and ~48 μM, respectively (Ge et al., 2011; Lott et al., 2011). K_d_ values in the low nanomolar range are difficult to reconcile with the finding that *in vivo *importin-α-mediated nuclear import cannot be saturated even by ~20-fold molar excess of NLS-cargo suggesting that the actual dissociation constants in the cytoplasm are significantly higher, possibly due to competitive binding of other cytoplasmic proteins to importin-α (Timney et al., 2006). Indeed, a non-invasive FRET/FLIM approach revealed K_d_ values in the low micromolar range in mammalian cells and substantiates the idea that formation of the NLS/importin-α complex in the cytoplasm is the rate-limiting event for nuclear import (Cardarelli et al., 2009). Artificial NLS peptides with extremely low K_ d_ values interfere with dissociation of the NLS/importin-α complex in the nucleus and prevent recycling of importin-α to the cytoplasm (Kosugi et al., 2008). Consequently, these peptides inhibit nuclear import. Whether some cargo proteins with high-affinity NLS such as the cap-binding complex remain bound to importin-α in the nucleus is still matter of discussion (Görlich et al., 1996b; Dias et al., 2009, 2010).

A significant number of host-targeted pathogen effector proteins localize entirely to host cell nuclei, indicating active nuclear import or passive diffusion through the NPC and sequestration in the nucleus (Deslandes and Rivas, 2011; Caillaud et al., 2012a,b). Generally, nuclear localization correlates with NLS motifs in the primary sequence suggesting that these effectors exploit the host cell’s nuclear import machinery for nuclear translocation. To what extent nuclear-targeted effectors need to compete with endogenous cargos is not understood. Effectors presumably act at relatively low protein levels to prevent activation of host defense. Given their low abundance and requirement for efficient nuclear targeting, effector NLSs might be an interesting source of high-affinity NLSs. Positioning effector NLSs within the above functional affinity limits will reveal whether pathogens evolved atypical NLS motifs that promote efficient nuclear import of effectors. Given the importance of nucleocytoplasmic transport for some immune pathways it has been hypothesized that microbial effectors might not only exploit but also manipulate or mimic components of the nuclear translocation machinery to subvert defense signaling. It is known that some animal viruses interfere with nucleocytoplasmic trafficking (Cohen et al., 2012), however, for microbial pathogens experimental proof for this hypothesis is lacking.

The reports discussed in this review substantiate the idea that tissue-specific expression, importin-α protein levels and sequence variation in the NLS binding cleft determine which importin-α functions as NTR for a cargo protein. However, more thorough analyses of plant NLS/importin-α complexes both *in vitro* and *in vivo* using emerging quantitative cell biology approaches are required to understand the complex regulation of nuclear import. Finally, many nuclear proteins do not have canonical NLS motifs. Although other import routes such as direct binding to importin-β (Marfori et al., 2011) or binding to other NTRs (Genoud et al., 2008) can account for some of these observations, the quest for novel NLSs continues.

## Conflict of Interest Statement

The authors declare that the research was conducted in the absence of any commercial or financial relationships that could be construed as a potential conflict of interest.
